# Peripherally Administered Botulinum Toxin Type A Localizes Bilaterally in Trigeminal Ganglia of Animal Model

**DOI:** 10.3390/toxins13100704

**Published:** 2021-10-05

**Authors:** Arief Waskitho, Yumiko Yamamoto, Swarnalakshmi Raman, Fumiya Kano, Huijiao Yan, Resmi Raju, Shaista Afroz, Tsuyoshi Morita, Daisuke Ikutame, Kazuo Okura, Masamitsu Oshima, Akihito Yamamoto, Otto Baba, Yoshizo Matsuka

**Affiliations:** 1 Department of Stomatognathic Function and Occlusal Reconstruction, Graduate School of Biomedical Sciences, Tokushima University, Tokushima 770-8504, Japan; arief.waskitho.85@gmail.com (A.W.); swar.raman@gmail.com (S.R.); jiaojiaole@hotmail.com (H.Y.); c000030613@tokushima-u.ac.jp (D.I.); okura.kazuo@tokushima-u.ac.jp (K.O.); m-oshima@tokushima-u.ac.jp (M.O.); 2 Department of Bacteriology, Okayama University Graduate School of Medicine, Dentistry and Pharmaceutical Sciences, Okayama 700-8558, Japan; yumiya@md.okayama-u.ac.jp; 3 Department of Tissue Regeneration, Graduate School of Biomedical Sciences, Tokushima University, Tokushima 770-8504, Japan; fkano@tokushima-u.ac.jp (F.K.); akihito@tokushima-u.ac.jp (A.Y.); 4 Department of Oral Disease Research, National Center for Geriatrics and Gerontology, Obu 474-8511, Japan; rajuresmi@ncgg.go.jp; 5 Department of Prosthodontics, ZA Dental College, Aligarh Muslim University, Aligarh 202002, India; shaista_afroz@yahoo.com; 6 Department of Oral and Maxillofacial Anatomy, Graduate School of Biomedical Sciences, Tokushima University, Tokushima 770-8504, Japan; morita.tsuyoshi@tokushima-u.ac.jp (T.M.); baba.otto@tokushima-u.ac.jp (O.B.)

**Keywords:** botulinum toxin, trigeminal ganglion, neuropathic pain

## Abstract

Peripheral nerve injury leads to sensory ganglion hyperexcitation, which increases neurotransmitter release and neuropathic pain. Botulinum toxin type A (BoNT/A) regulates pain transmission by reducing neurotransmitter release, thereby attenuating neuropathic pain. Despite multiple studies on the use of BoNT/A for managing neuropathic pain in the orofacial region, its exact mechanism of transport remains unclear. In this study, we investigated the effects of BoNT/A in managing neuropathic pain in two different animal models and its transport mechanism in the trigeminal nerve. Intraperitoneal administration of cisplatin induced bilateral neuropathic pain in the orofacial region, reducing the head withdrawal threshold to mechanical stimulation. Unilateral infraorbital nerve constriction (IONC) also reduced the ipsilateral head withdrawal threshold to mechanical stimulation. Unilateral peripheral administration of BoNT/A to the rat whisker pad attenuated cisplatin-induced pain behavior bilaterally. Furthermore, contralateral peripheral administration of BoNT/A attenuated neuropathy-induced behavior caused by IONC. We also noted the presence of BoNT/A in the blood using the mouse bioassay. In addition, the Alexa Fluor-488-labeled C-terminal half of the heavy chain of BoNT/A (BoNT/A-Hc) was localized in the neurons of the bilateral trigeminal ganglia following its unilateral administration. These findings suggest that axonal and hematogenous transport are involved in the therapeutic effects of peripherally administered BoNT/A in the orofacial region.

## 1. Introduction

Nerve injury in the orofacial region—particularly involving the trigeminal nerve—instigates prolonged pain throughout the surrounding area [1]. The interactions between peripheral and central mechanisms are responsible for the persistence of pain linked with the trigeminal nerve [1]. Peripheral nerve injury leads to neuropathic pain and sensory ganglion hyperexcitation [2]. Previous studies have reported that the hyperexcitation of sensory ganglion neurons increases neurotransmitter release [3,4,5]. Previous studies on the application of botulinum neurotoxin type A (BoNT/A) have demonstrated its effectiveness in reducing neuropathy-induced behavior, which indicates that neurotransmitter release in the sensory ganglia is involved in the regulation of pain transmission [6,7,8].

Botulinum neurotoxin (BoNT), which is produced by *Clostridium botulinum*, is widely known for its use in cosmetic treatments. Based on serological type, BoNT can be classified into seven categories: BoNT/A–G. In the management of neurological disorders, the most widely used type is BoNT/A because it has long-lasting effects [9]. The structure of BoNT is made up of polypeptide chains; namely, a light (L) chain (50 kDa) and a heavy (H) chain (100 kDa) that are connected by a disulfide bond. The H chain has a receptor-binding domain (the C-terminal half of the heavy chain (Hc)), which binds to proteins and gangliosides on the cell surface [10], as well as a translocation domain (the N-terminal half of the heavy chain (Hn)). The L chain cleaves specific proteins, such as vesicle-associated membrane protein (VAMP), synaptosomal-associated protein 25kDa (SNAP-25), and syntaxin [10].

Several previous studies have reported the efficiency of BoNT for inhibiting the release of neurotransmitters, including acetylcholine, glutamate, noradrenaline, serotonin, substance P, calcitonin gene-related peptide (CGRP), adenosine triphosphate, and nicotinamide adenine dinucleotide (NAD) [10,11,12,13,14]. 

The effectiveness of BoNT/A for managing neuropathic pain is also related to its distribution beyond the injection site. The results of our previous study, in which BoNT/A was injected into the whisker pad area, suggest that BoNT/A reaches the trigeminal ganglion (TG) to decrease an exaggerated neurotransmitter release [7]. Moreover, intramuscular injection of BoNT/A results in its axonal transport to the spinal cord via transcytosis; these findings suggest a novel pathway for BoNT/A after peripheral application [15]. The concept of axonal transport of BoNT in the central neurons was first proposed by Antonucci et al., and the long-distance retrograde effects were discussed in the visual system [16]. However, to date, no study has demonstrated the concept of axonal transport in conditions of orofacial neuropathic pain.

In the present study, we investigated the effects of peripheral administration of BoNT/A in the treatment of neuropathic pain, and evaluated its transportation in the trigeminal nerve.

## 2. Results

### 2.1. Head Withdrawal Threshold after Chemotherapy-Induced Bilateral Neuropathy and BoNT/A Application 

**Figure 1:**
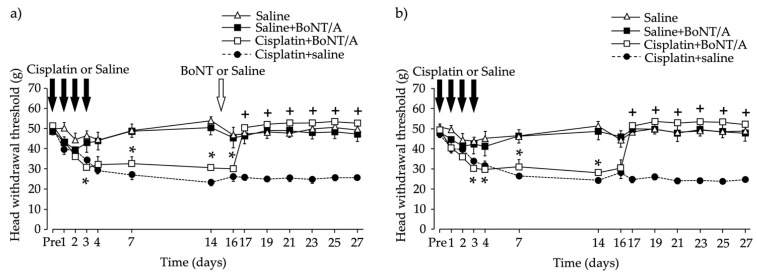
Head withdrawal thresholds after cisplatin administration and unilateral peripheral botulinum toxin type A (BoNT/A) application into the whisker pad. (**a**) Ipsilateral (injection side); (**b**) contralateral (no injection). Cisplatin administration reduced the head withdrawal threshold, and intradermal application of BoNT/A into the whisker pad 15 days after the first cisplatin intraperitoneal injection (10 minimal lethal dose in 100 μL saline) increased the threshold to the baseline level. The head withdrawal thresholds to mechanical stimuli are shown as the mean ± standard error of the mean. * *p* < 0.05, Cisplatin + BoNT/A group vs. Saline group. + *p* < 0.05, Cisplatin + BoNT/A group vs. Cisplatin group (repeated measures analysis of variance with Bonferroni *p*-value adjustment). *n* = 6 per group.

The baseline mechanical stimuli sensitivity of each rat was recorded, and cisplatin was then intraperitoneally injected for four consecutive days. Sensitivity to mechanical stimuli was increased bilaterally in the whisker pad area (Figure 1). The head withdrawal threshold baseline for the Cisplatin + BoNT/A group was 51.5 ± 0.45 g for the ipsilateral side and 49.3 ± 0.92 g for the contralateral side. The threshold significantly decreased, to approximately 30.7 ± 3.83 g for the ipsilateral side (Figure 1a) and 30.1 ± 4.29 g for the contralateral side (Figure 1b), three days after the first intraperitoneal cisplatin injection. Unilateral intradermal injection of BoNT/A into the whisker pad restored the head withdrawal threshold to the baseline level bilaterally (Figure 1). The BoNT/A effect was observed two days after application and lasted for the next 12 days.

### 2.2. Head Withdrawal Threshold after Infraorbital Nerve Constriction (IONC) and Contralateral BoNT/A Application

**Figure 2:**
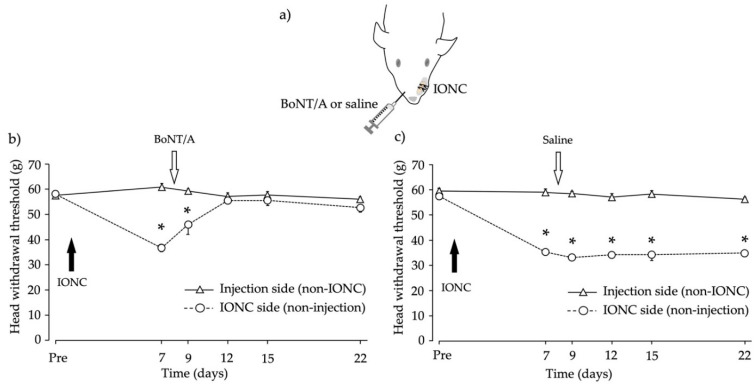
Behavioral changes after the peripheral administration of botulinum toxin type A (BoNT/A) or saline to the contralateral side of infraorbital nerve constriction (IONC) rats. (**a**) Schematic illustrating IONC and the BoNT/A injections. (**b**) BoNT/A application into the contralateral whisker pad increased the withdrawal threshold to mechanical stimulation. There were no significant differences between the BoNT/A-injected side and the IONC side on days 12, 15, or 22 after IONC. (**c**) Injection of saline did not increase the withdrawal threshold to mechanical stimulation. There was a significant difference between the saline-injected side and the IONC side on days 7, 9, 12, 15, and 22 after IONC. The head withdrawal thresholds to mechanical stimuli are shown as the mean ± standard error of the mean. * *p* < 0.05, BoNT/A- or saline-injected side vs. IONC side (repeated measure analysis of variance with Bonferroni *p*-value adjustment). *n* = 6 per group.

The experimental design is shown in Figure 2a. IONC decreased the head withdrawal threshold on the IONC side. The baseline withdrawal threshold for the IONC side was 58.1 ± 1.12 g for the BoNT/A-injected group (Figure 2b) and 57.6 ± 0.76 g for the saline-injected group (Figure 2c). In the BoNT/A-injected group, the withdrawal threshold was markedly reduced to 36.7 ± 1.56 g at 7 days after IONC. Four days after BoNT/A application, the withdrawal threshold was increased to near the baseline level; this effect lasted for 10 days (Figure 2b). In contrast, in the saline-injected group, the withdrawal threshold decreased to 35.3 ± 1.19 g; this effect did not recover and lasted for 15 days (Figure 2c).

### 2.3. Mouse Bioassay to Detect BoNT/A Concentrations in the Blood

BoNT/A (5000 minimal lethal dose (MLD)) was injected intradermally into the whisker pad of a rat. Five hours later, a cardiac puncture was performed to collect blood, and the serum was separated. The serum was then diluted and injected intraperitoneally (0.5 mL) into mice. Because the mice in the 50× dilution group survived, we calculated that the BoNT/A concentration at 20× dilution was 40 MLD/mL (Table 1 A and B).

Ideally, to evaluate the total concentration of BoNT/A in the blood, we require the total blood volume of the rat. However, acquiring total blood volume using cardiac puncture is nearly impossible; hence, we calculated the total blood volume using an experimentally determined equation proposed by a previous study [17]. Total rat blood volume was calculated in relation to body weight. Because the rat body weight was 360 g, the estimated blood volume was 22.37 mL. Thus, the total estimated concentration of BoNT/A in the blood was calculated as 894.8 MLD (approximately 18% of 5000 MLD) (Table 1C). 

### 2.4. Peripherally Administered BoNT/A-Hc Localized in the TG

**Figure 3:**
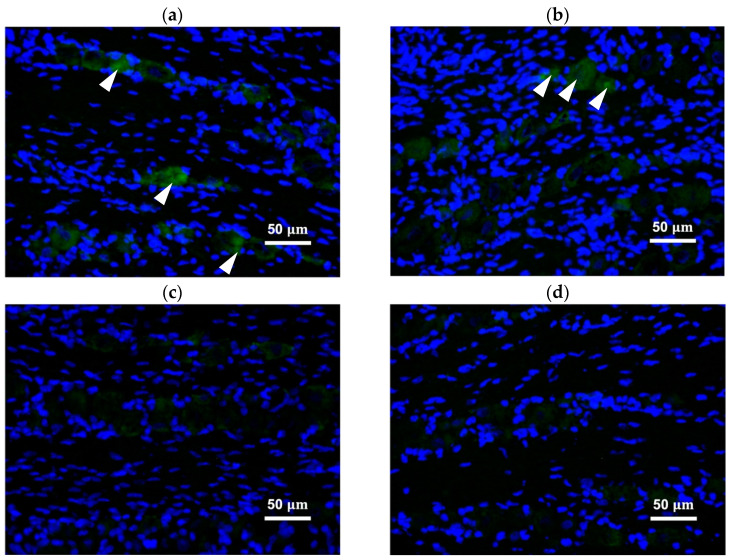
BoNT/A-Hc labeled with Alexa Fluor-488 was localized in the trigeminal ganglion V2 area. (**a**,**b**) Fluorescence micrographs showing BoNT/A-Hc labeled with Alexa Fluor-488 (green) co-localized with NucBlue (Hoechst 33342)-stained nuclei (blue), in the (**a**) ipsilateral side and (**b**) contralateral side. (**c**,**d**) Fluorescence micrographs showing the solvent liquid with Alexa Fluor-488 only (control) administered into the whisker pad, in the (**c**) ipsilateral side and (**d**) contralateral side. Arrowheads indicate the localization of BoNT/A-Hc labeled with Alexa Fluor-488 in neurons. Scale bar = 50 μm.

**Figure 4:**
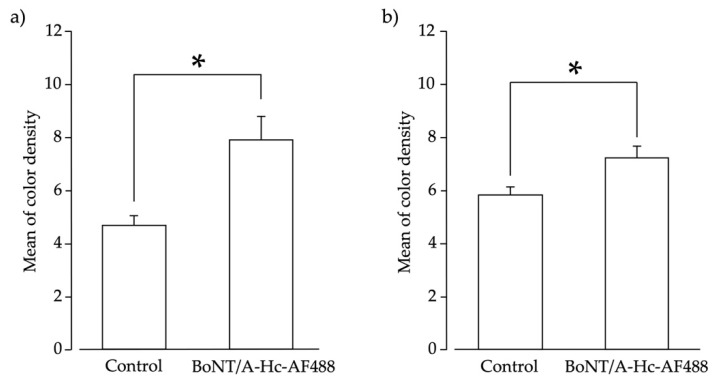
Densitometric analysis of neurons of the trigeminal ganglion (TG). The mean color density of BoNT/A-Hc labeled with Alexa Fluor-488 was measured in neurons in the TG V2 area on both the (**a**) ipsilateral side and (**b**) contralateral side. The optical densities of the TG neurons in rats treated with Alexa Fluor-488 only (control; *n* = 3) and BoNT/A-Hc-Alexa Fluor-488 (BoNT/A-Hc-AF488; *n* = 3) are shown. * *p* < 0.05 (*t*-test).

The C-terminal half of the heavy chain of BoNT/A (BoNT/A-Hc) labeled with Alexa Fluor-488 was peripherally administered into the whisker pad to confirm its bilateral transportation. Neurons in the TG were recognized as the nucleus (located in the center of the neuron), and were surrounded by satellite glial cells. BoNT/A-Hc labeled with Alexa Fluor-488 was localized in neurons of the bilateral TG V2 area (Figure 3a,b) four days after its peripheral injection into the whisker pad. We also measured the color density of BoNT/A-Hc labeled with Alexa Fluor-488 localized in the nucleus, and compared the results with the control group. (Figure 4a,b). The mean color density measurement of BoNT/A-Hc labeled with Alexa Fluor-488 in the bilateral TG V2 area was significantly higher than that of Alexa Fluor-488 only (*p* < 0.05, *t*-test).

## 3. Discussion

BoNT, and especially BoNT/A, has been widely used to treat neurological conditions in recent decades. The conditions wherein BoNT/A has been explored include complex regional pain syndrome, postherpetic neuralgia, trigeminal neuralgia, diabetic neuropathy, and occipital neuralgia [18]. Several clinical studies have also suggested the effectiveness of BoNT/A for treating trigeminal neuralgia [19,20,21,22]. Additionally, chronic constriction injury models have been studied in the recent past to better understand the mechanism of the effectiveness of BoNT/A [23,24,25]. A recent literature review of BoNT and cancer suggested that the local administration of BoNT aids in the treatment of post-radiation or post-surgical pain [26]. Despite extensive clinical and animal research on the analgesic effects of BoNT in neuropathic pain, uncertainty remains about its exact mechanism of action. The current hypothesis involves either the inhibition of neurotransmitter release or the retrograde axonal transport of BoNT [10,11,12,13,14,15]. In the present research, we used peripherally administered BoNT/A to study the attenuation of pain in neuropathic pain models, and demonstrated the transmission of BoNT/A in sensory neurons of the TG.

The main findings of the current study were as follows: (1) unilateral peripheral injection of BoNT/A increased the head withdrawal threshold bilaterally in a chemotherapy-induced neuropathic model, (2) peripheral injection of BoNT/A on the contralateral side increased the head withdrawal threshold in an IONC model, (3) BoNT/A was present in the blood following intradermal injection, and (4) after unilateral peripheral administration, BoNT/A-Hc was localized in neurons of the bilateral TG.

Cisplatin-induced peripheral neuropathy is one of the most common causes of referral to pain management clinics. Cisplatin induces sensory neuropathy, which manifests as distal paresthesia, sensory ataxia, dorsal column myelopathy, and even bilateral jaw pain [27]. Despite numerous studies on possible pharmacological therapeutic interventions to treat or alleviate chemotherapy-induced neuropathic pain, no effective treatments exist to date [28]. Previous animal research using the paclitaxel-induced neuropathic pain model has demonstrated reduced hypersensitivity in the hind-paw region after the subplantar injection of BoNT/A [29]. In our study, the pain threshold to mechanical stimuli was altered in the chemotherapy-induced neuropathy model after the peripheral injection of BoNT/A in the whisker pad area.

In previous animal model studies, the use of BoNT/A has been reported to induce sensorimotor recovery in the injured peripheral nerve [30]. The intraplantar administration of BoNT/A strongly reduces both mechanical and thermal hyperalgesia and accelerates the recovery from injury [31]. Furthermore, in an experimental diabetic neuropathy model, unilateral BoNT/A injection led to bilateral reduced thermal and mechanical hypersensitivity [32]. Similarly, the ipsilateral injection of BoNT/A in a trigeminal neuralgia model was reported to increase the threshold for mechanical stimulation [24]. In the present study, we used an IONC model, and the peripheral administration of BoNT/A (contralaterally to the nerve injury) led to alleviated pain behavior in the whisker pad area on the IONC side. This BoNT/A effect was suggested to involve retrograde axonal transport via the neurons, which is also in accordance with the hypotheses of previous reports [31,32].

In the current study, unilateral BoNT/A injection alleviated chemotherapy-induced neuropathic pain bilaterally, and contralateral BoNT/A injection alleviated pain behavior with IONC. These findings support the results of previous studies, indicating that peripheral administration of BoNT/A is effective for treating chemotherapy-induced neuropathy and neuropathic pain in the orofacial region [15,24,32]. BoNT/A transmission via the bloodstream and/or neural pathways has been suggested previously [15,33]. 

We also investigated the blood and neural transmission of BoNT/A. The gold standard for the detection and confirmation of BoNT in the blood is the in vivo mouse bioassay. This is a highly sensitive, robust, and semiquantitative technique [34,35]. In the present study, serial dilutions of the assay were performed; only three of the mice were free of symptoms, whereas the few who survived showed classical “wasp-waist” symptoms. In our experiment, the last death of mice in the mouse bioassay occurred at 20× dilution (per 0.5 mL; intraperitoneal injection), which indicates that the concentration of BoNT/A in the blood was 40 MLD per mL. Our findings suggest that approximately 18% of the intradermally injected BoNT/A in the rat whisker pad was transported to the bloodstream. Previous studies have also demonstrated the bilateral transmission of a unilateral intramuscular injection of BoNT/A via the blood using measurements of compound muscle action potentials [15]. Together, these results indicate that BoNT/A is transported through the bloodstream beyond the peripheral site of application. Although BoNT/A transports through blood circulation, it does not cause systemic adverse effects such as motor weakness, as proven in our previous study [6]. Similarly, a prior study that injected BoNT/A into the whisker pad reported no impairment in motor coordination [24].

To interpret the bilateral neural transmission, we also studied the localization of BoNT/A-Hc beyond the injection site. We observed BoNT/A-Hc labeled with Alexa Fluor-488 in neurons of the bilateral TG after a unilateral peripheral injection. Several studies have also reported the similar concept of transcytosis, but evidence is lacking for the precise mechanism underlying this effect [11,15,29,32]. In a previous model of trigeminal neuralgia, subcutaneous BoNT/A was administered, and transient receptor potential (TRP) channels and SNAP-25 were analyzed to confirm the antinociceptive effect of BoNT/A associated with axonal transport [24]. To the best of our knowledge, the present study is the first to report the bilateral localization of BoNT/A in the TG after its unilateral peripheral administration. 

The bilateral transport of BoNT/A-Hc labeled with Alexa Fluor-488 to neurons in the V2 area of the TG corroborated our primary finding in the neuropathic pain models, in which the unilateral peripheral administration of BoNT/A attenuated pain bilaterally. These findings are also complementary to the observation of cleaved SNAP-25 in the spinal cord after intramuscular injection of BoNT/A in a previous study [15]. Our findings support the occurrence of cell-to-cell trafficking, which may be the mechanism underlying the bilateral localization of BoNT/A-Hc in the TG.

Previous research into the peripheral administration of BoNT/A in trigeminal neuropathy models has demonstrated the role of neurotransmitter release from sensory neurons in the TG [7]. Furthermore, several other studies of chronic constriction injury models have reported an analgesic effect of BoNT/A, and have hypothesized the role of axonal transport in this effect [7,8,24,25]. In the current study, rather than only considering the possibility of BoNT/A transport mechanisms, we demonstrated the bloodstream circulation of BoNT/A and revealed its localization bilaterally in the TG after unilateral administration. These findings suggest that hematogenous as well as axonal transport is involved in the therapeutic effects of BoNT/A. Nonetheless, further studies are required to demonstrate the contribution of central mechanisms to peripheral pain attenuation. Research on the whole brain, including the brainstem, may aid in understanding the central synapse after axonal transport and transcytosis within the trigeminal complex.

## 4. Conclusions

In the present study, we revealed that peripheral application of BoNT/A to the whisker pad restored nocifensive parameters bilaterally in rat models of chemotherapy-induced neuropathic pain and IONC. We also demonstrated that hematogenous transport was involved in the therapeutic effects of BoNT/A, and that unilaterally administered BoNT/A was localized in the neurons of the bilateral TG. These findings point to the involvement of axonal and hematogenous transport in the therapeutic effects of peripherally administered BoNT/A in the orofacial region.

## 5. Materials and Methods

### 5.1. Animals

Male Sprague Dawley rats (weighing 120–360 g; CLEA Japan, Osaka, Japan) were used in these experiments. The animals were kept in a controlled animal room (19–21 °C and 12 h light/dark cycle) and animals were fed ad libitum with a regular diet and allowed free access to water. 

All surgical procedures were performed under anesthesia using 0.375 mg/kg medetomidine (Nippon Zenyaku Kogyo Co., Ltd., Fukushima, Japan), 2 mg/kg midazolam (Sandoz K.K., Yamagata, Japan), and 2.5 mg/kg butorphanol (Meiji Seika Pharma Co., Ltd., Tokyo, Japan).

### 5.2. Chemotherapy-Induced Bilateral Neuropathic Pain

Chemotherapy-induced neuropathy was induced by intraperitoneal injection of cisplatin (2 mg/kg/day; Yakult Co., Ltd., Tokyo, Japan), administered daily for 4 consecutive days. The control animals were injected with saline. 

### 5.3. IONC

The IONC surgery was performed intraorally as described in previous studies [36,37], which allows the whisker pad to remain intact. Thus, the surgery did not interfere with behavioral testing. After behavioral training and recording of the baseline, the IONC surgery was performed. Rats were deeply anesthetized, and an incision was made in the hard palate proximal to the first molar extending approximately 1 cm anteriorly (parallel to the lip). The nerve was exposed, cleared from the surrounding tissue, and loosely tied with two ligatures (4–0 silk suture). The wounds were then closed with veterinary tissue adhesive (Vetbond, 3M Animal Care Products, St. Paul, MN, USA).

### 5.4. Drug Administration

Purified BoNT/A was prepared according to previous studies [38]. Under general anesthesia, the rats were injected with purified BoNT/A (10 MLD in 100 μL saline) or saline, which was intradermally administered to one side in the center of the whisker pad (i.e., between rows B and C of the vibrissae). For the chemotherapy-induced bilateral neuropathic pain model animals, purified BoNT/A was injected on day 15 after the first cisplatin injection, and for the IONC pain model animals, purified BoNT/A was injected on day 8 after surgery. 

### 5.5. Behavioral Testing

One day before behavioral testing, each rat’s whiskers were shaved bilaterally at the orofacial region using clippers. Each rat was restrained in a holder (Durham Holders, 37100, Ugo Basile, Varese, Italy) with a small semicircular hole (diameter: 7 cm, height: 2 cm) that allowed their snout to protrude from the holder and left their head unrestricted during stimulation. Mechanical stimuli were applied at the center of the whisker pad (i.e., between rows B and C of the vibrissae) using an electronic von Frey anesthesiometer (Model 1601C, IITC Instruments, Woodland Hills, CA, USA) until the animal withdrew its head. Stimuli were applied on both the ipsilateral and contralateral sides. The force (g) that was applied at the time of head withdrawal was recorded. Each side was recorded five times with 1-min intervals, and the results were averaged after eliminating the lowest and highest values. 

Behavioral testing was conducted before the injection of anticancer medication drugs for the baseline, and on days 1, 2, 3, 4, 7, and 14 after the anticancer medication administration. The behavioral testing was repeated on days 16 and 17 after BoNT/A injection and repeated every two days until day 27. Behavioral testing for the IONC pain model was performed 1 day before surgery for the baseline, and on days 7, 9, 12, 15, and 22 after surgery. All behavioral assessments were performed by an examiner who was blind to the injection materials and sides.

### 5.6. Mouse Bioassay

The mouse bioassay was performed to evaluate whether BoNT/A was transmitted through blood circulation. A rat was deeply anesthetized and intradermally injected with 5000 MLD of purified BoNT/A in the whisker pad. Five hours later, blood was collected by cardiac puncture as described previously [39], using a 5 mL syringe with a 23 G needle. The blood was allowed to clot at room temperature for about 30–60 min and was then centrifuged for 20 min at 1300× *g* at 25 °C. Serial dilution of the serum was performed using 20 mM sodium phosphate buffer (pH 6.0) containing 0.2% (weight/volume) gelatin, and 0.5 mL of each dilution was then intraperitoneally injected into female ICR mice (weighing 21–27 g). The mice were observed for 1 week, and the MLD per mL was measured. Total BoNT/A concentration in the blood was calculated using the following equation:

BoNT/A concentration/mL × blood volume

where blood volume was calculated using an equation proposed by a previous study [17]:

Blood volume = 0.06 × body weight + 0.77

### 5.7. Immunohistochemistry

For histological studies, naïve rats were used. To visualize the BoNT/A-Hc, we used recombinant BoNT/A-Hc that was labeled with Alexa Fluor-488 maleimides according to previous studies [6]. Rats were deeply anesthetized and either BoNT/A-Hc labeled with Alexa Fluor-488 (10 μg/100 μL) or Alexa Fluor-488 maleimides (20 μM) only (control) was administered intradermally using a 28 G × 13 mm × 0.5 mL syringe. At 4 days post-injection, all rats were deeply anesthetized and the bilateral TG were dissected. The tissue was embedded in Tissue-Tek optimal cutting temperature compound (Agar Scientific, Stansted, UK) and flash frozen in liquid nitrogen. Frozen sections of 10 μm thickness were cut on a cryostat (CM1850S, Leica, Wetzlar, Germany) and mounted onto silane-coated slides (Matsunami, Osaka, Japan). The slides were coverslipped using ProLong Glass Antifade Mountant with NucBlue (Hoechst 33342; Thermo Fisher Scientific, Life Technologies Corp., Eugene, OR, USA). Fluorescence images were acquired on a Keyence BZ-X800 All-in-One Fluorescence Microscope (Keyence, Osaka, Japan) and analyzed with ImageJ software (NIH, Bethesda, MD, USA).

### 5.8. Densitometric Analysis

To evaluate the approximate density of BoNT/A-Hc labeled with Alexa Fluor-488, images of the V2 area of TG sections from rats that received BoNT/A-Hc labeled with Alexa Fluor-488 or Alexa Fluor-488 (control) were simultaneously acquired using the same protocols. Using ImageJ software, the mean color densities of immunostaining products were measured in the raw digital images of the V2 area of the TG. For each animal that received BoNT/A-Hc labeled with Alexa Fluor-488 (*n* = 3) or Alexa Fluor-488 (control) (*n* = 3), measurements were made specifically in the neuronal area.

### 5.9. Statistical Analysis

Data are presented as the mean ± standard error of the mean. Statistical analysis was performed using repeated measures analysis of variance followed by the Bonferroni *p*-value adjustment method or *t*-test, using SPSS 27 software (IBM, Tokyo, Japan). *p* < 0.05 was considered significant. 

## Figures and Tables

**Table 1:** Mouse bioassay of BoNT/A.

**BoNT/A Injection**		**Dilution**					
		2×	5×	10×	20×	50×	100×
A	Peripheral injection	××	××	×△×	××	○△	○○
B	BoNT/A concentration/0.5 mL = 20 MLD BoNT/A concentration/mL = 20 MLD X 2 = 40 MLD						
C	Blood volume = 0.06 × body weight + 0.77 = 0.06 × 360 + 0.77 = 22.37 mL Total BoNT/A concentration in blood = 40 MLD X 22.37 mL = 894.8 MLD						

×: death; △: symptoms (survival); ○: no symptoms; BoNT/A: botulinum toxin type A; MLD: minimal lethal dose. ICR mice weighing 21–27 g were used.

## Data Availability

The datasets generated and analyzed during the current study are available from the corresponding author on reasonable request.

## Institutional Board Review

The study was conducted according to the National Institutes of Health Guide for the Care and Use of Laboratory Animals. All protocols were approved by the Ethics Committee of Tokushima University (protocol numbers: T30-13, approval date 1 May 2018; and T2020-108, approval date 1 May 2021).
